# Experimental study on cement mortar with magnetized water treated by composite time-varying electromagnetic fields

**DOI:** 10.1038/s41598-025-24787-x

**Published:** 2025-11-07

**Authors:** Wu Zhao, Jinliang Wang, Tao Li, Chang Ni

**Affiliations:** 1 Key Laboratory for Road Construction Technology and Equipment of Ministry of Education, School of Construction Machinery, Chang’ an University, Xi’an, 710064 People’s Republic of China; 2 School of Construction Machinery, Chang’ an University, Xi’an, 710064 People’s Republic of China

**Keywords:** Composite time-varying electromagnetic field, Magnetic field frequency, Compressive strength, Magnetized water, Cement mortar, Engineering, Materials science

## Abstract

The present study investigated the effects of composite time-varying electromagnetic fields on the microstructure and macroscopic mechanical properties of cement-based composites. Fourteen magnetized water samples were prepared using direct current (DC), sinusoidal/triangular/rectangular alternating currents (AC) and their combined electromagnetic field configurations. Comprehensive characterization was conducted to elucidate the evolution of physicochemical properties and their subsequent effects on the compressive strength of M20 cement mortar. The experimental findings demonstrated that critical parameters of magnetized water, the maximum increases in pH value, conductivity and viscosity of water, were 30.9%, 32.3% and 24.3% respectively. Conversely, surface tension displayed an inverse relationship with frequency elevation. Studies have shown that the application of magnetized water treatment technology can significantly enhance the compressive strength of cement mortar. When treated with a DC-sinusoidal combined electromagnetic field of 5 kHz, the 7d compressive strength of cement mortar can reach a peak of 19.39 MPa. This strength value represents an 11.4% increase compared to the control group. The DC-triangular combined electromagnetic field (1 kHz) exhibited a substantial 32.5% enhancement in 28d compressive strength. A comparative analysis was conducted, which confirmed that the synergistic effects of combined time-varying electromagnetic fields were superior to those of individual magnetic field applications. These findings provided significant implications for optimizing electromagnetic processing parameters to improve the performance of cementitious materials and for construction material engineering.

## Introduction

As our understanding of the fundamental nature of magnetic phenomena has gradually evolved, there has been an increasing interest in exploring their practical applications across a range of disciplines. The utilization of magnetic fields in the treatment of water constituted a significant research trajectory. The activation of water by magnetic fields is the core of this process, with the objective being the regulation of the hydrogen bonding network structure of water through the utilization of magnetic field energy. In liquid water, water molecules generally exist in large quantities in the form of clusters, and multiple water molecules are connected by hydrogen bonds to form macromolecular structures^1–3^. The magnetization process facilitates the conversion of metastable macroclusters into stable subcluster structures^4–6^. Magnetic field-induced structural changes significantly altered water properties, including a decrease in surface tension and an enhancement in ionic dissolution capacity (conductivity assays). These changes endowed the water with novel characteristics and application potential^7–9^.

The utilization of magnetic water activation technology signified a pioneering physical water treatment approach, which evinced a distinctive potential in the domain of cementitious material modification. Contemporary methodologies predominantly encompassed two technological methods: permanent magnet-based static fields and electromagnet-driven time-varying fields. Permanent magnet systems employed hard magnetic materials (e.g. NdFeB) to generate static magnetic fields. These systems offered the advantages of structural simplicity and low energy consumption, which had facilitated their widespread adoption in practical engineering. Demir^10^ noted incorporating CMP and magnetized water (MW) in AAS mortars further improved fresh and hardened state properties compared to RMP and tap water (TW). Hamed^11^ showed that compared with TW, utilizing MW in preparing the AA of geopolymer concrete caused notable increase in the concrete slump with an average of 14%, compressive strength increase by up to 64%, tensile strength increase by up to 60%, flexural strength increase by up to 41%.

Hu^12^ from Central South University engineered a multi-layered permanent magnet array. The resultant cement mortar, mixed with magnetized water and processed by this configuration, exhibited enhanced consolidation and compressive strength. Wang Youkai’s group innovated with an adjustable-interval permanent magnet assembly, achieving a 24.8% increase in 28d compressive strength of magnetized water-mixed concrete through systematic optimization of magnetic flux parameters^13^. Ghorbani^14^ employed a hollow cylindrical magnetization device, reporting a 74% enhancement in the stability of foam concrete incorporating magnetized water, thereby validating the scalability of static-field applications.

Electromagnetic induction technology produces a dynamic and controllable magnetic field through components such as electromagnetic coils. Yesin^15^ (ETH Zürich) realized continuous field intensity modulation through coil spacing adjustmen. Jeong’s team^16^ synthesized hybrid magnetic environments by superimposing rotational and static fields. Sikorski^17^ pioneered the BigMag system, enabling three-dimensional gradient field generation via coordinated electromagnetic coil positioning.

Despite these technological advancements, persistent challenges have been observed across both implementation methods. Permanent magnet systems are constrained by inherent limitations. These include non-adjustable flux density and irreversible annual demagnetization rates that exceed 3%. Electromagnetic field methods are primarily confined to single-frequency/waveform effects, with an absence of reasonable analysis on the multi-frequency coupling mechanisms (e.g., DC bias-high frequency AC superposition). This limitation hinders industrial applications. Further exploration and research in this area was required.

The principal research components included a multi-coil electromagnetic treatment system with tunable waveform superposition capabilities. A standardized testing platform was constructed for the systematic evaluation of magnetized water properties under diverse electrical signal configurations. The properties included pH variation, conductivity variation, surface tension reduction and viscosity alteration. Compressive performance assessments of cement mortar specimens were performed, complemented by microstructural characterization through scanning electron microscopy (SEM).

## Experimental design

All test water was left to stand at room temperature (25 ± 1 °C) for 24 h to allow chlorine to volatilize naturally, thereby reducing the impact of chlorine on the experimental results. After that, it was circulated through the magnetization device via a water pump. In accordance with standardized protocols, the physicochemical parameters of the treated water—including pH, electrical conductivity, surface tension, and viscosity—were tested and recorded. The magnetized water was utilized as the medium for the preparation of cement mortar. Cubic mortar specimens measuring 70 × 70 × 70 mm were cast, and compressive strength testing was performed at the 7d and 28d curing stages using a servo-hydraulic universal testing machine. A microstructural analysis was conducted using scanning electron microscopy (SEM) in order to characterize the morphology and evolution of the hydration products.

### Preparation of the magnetized water

#### Magnetized water device

**Fig. 1:**
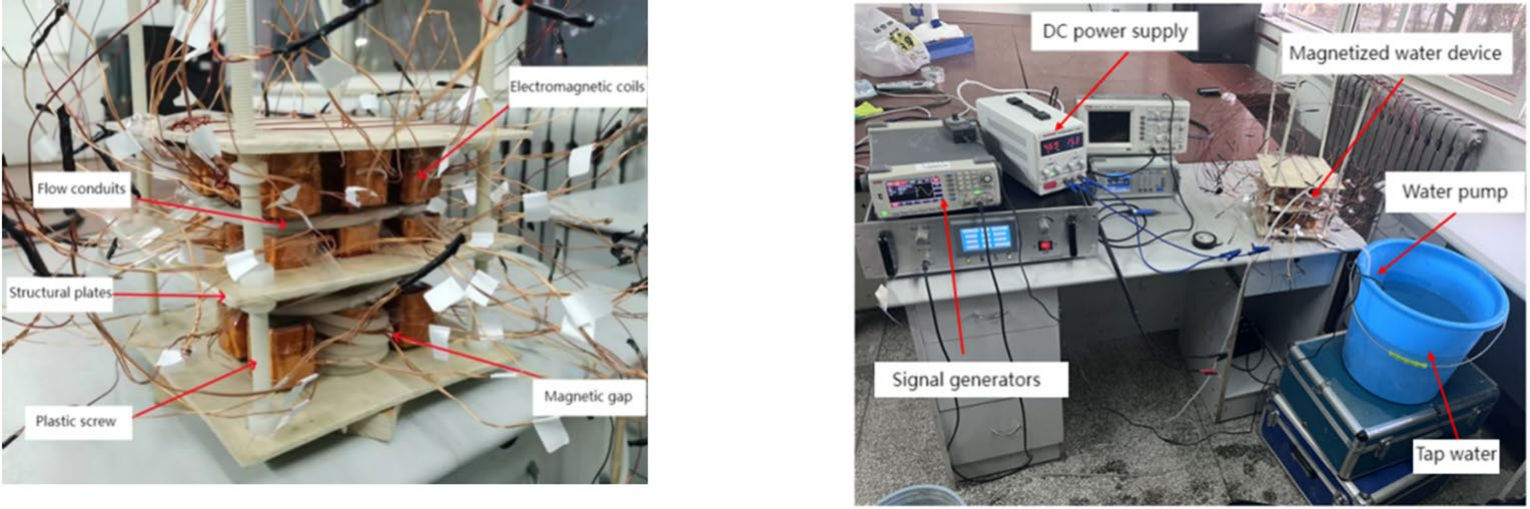
Schematic diagram of the magnetic field-activated water device structure and the magnetized water system.

**Fig. 2:**
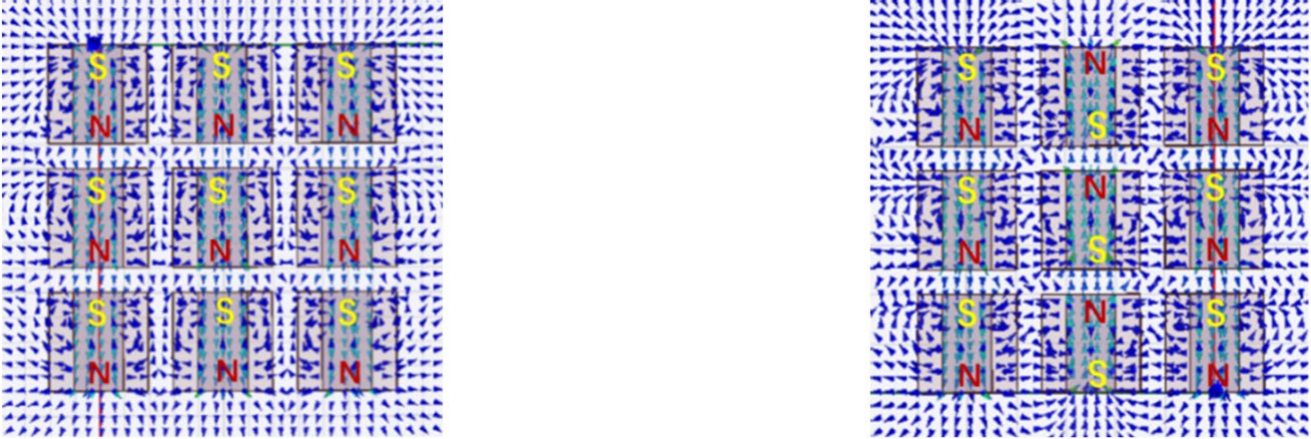
Schematic diagram of magnetic force lines under different arrangements of coils.

This study employed a custom-developed composite time-varying electromagnetic field activation system, as schematically depicted in Fig. 1. The apparatus integrated electromagnetic coils, structural plates, flow conduits, and plastic screws. The electromagnetic coil was equipped with a 20 mm× 20 mm× 40 mm rectangular cross-section ferromagnetic core, the purpose of which is to enhance the magnetic induction intensity. A 3D orthogonal array configuration (3 × 3 × 3 matrix) of 27 identical electromagnetic coils was implemented through hierarchically nested architecture, achieving optimized magnetic flux spatial distribution. The system delivered peak magnetic flux density of 455.52 mT (at an 8 kHz excitation frequency) with a spatial uniformity of less than 5%. The interlayer spiral water flow channel of the electromagnetic coil has been designed to ensure that the water flows through the device in the water pipe. Two distinct coil arrangement methods were prototyped based on electromagnetic field superposition principles (Fig. 2):

(i) Parallel alignment - All coil elements maintain identical magnetic polarization vectors, generating cumulative field reinforcement;
(ii) Star configuration - The adjacent columns are arranged in different directions.

#### Test system

The experimental configuration, as schematically represented in Fig. 1, comprised an AC signal generator, a DC power supply, power amplifier, water pipe, water pump, and a magnetic field-treated water (MFTW) device. A water flow rate of 0.6 m/s was maintained throughout the entire water treatment phase.

Following system activation, the water was subjected to continuous circulation through the electromagnetic treatment unit. In accordance with the extant research results and references of the research group, a standardized circulation duration of 80 min has been determined. The magnetized water be distributed to analytical instrumentation within five minutes of process termination to mitigate property decay. A total of 33 samples of magnetized water were prepared. All physicochemical measurements were systematically recorded.

#### Setting of test parameters

The experimental protocol incorporated three distinct alternating current waveforms (sinusoidal, triangular, and rectangular) that were methodically implemented. The frequency modulation of the applied magnetic field demonstrated to exert a significant influence on the efficacy of aqueous magnetization, as evidenced by prior studies^18,19^. The enhanced energy transfer efficiency was achieved through the following mechanisms: firstly, an increase in the frequency of magnetic field cycling, and secondly, an intensification of water molecular restructuring through repeated Lorentz force interactions^20,21^.

The magnetic field strength and power fluctuated violently in the range of 0 to 1 kHz. However, the variation decreased in the range of 1 kHz to 8 kHz. A frequency spectrum spanning 1 kHz to 8 kHz was systematically investigated under controlled waveform conditions. The purpose of this investigation was to elucidate the tripartite relationship between magnetic field frequency, current waveform typology, and magnetization performance. Precise waveform parameters (amplitude: 6 V; frequency range: 0–8 kHz) were programmatically controlled through a LabVIEW-based interface. Real-time magnetic flux density monitoring was synchronized through LabVIEW-integrated data acquisition, utilizing a high-precision Teslameter with a measurement uncertainty of ± 0.1%. This monitoring process ensured temporal waveform fidelity with harmonic distortion rates below 2%. As demonstrated in Table 1, the electrical signal configurations are meticulously catalogued.

**Table 1:** Electrical signal parameters.

Signal type	DC	Sine wave	Triangular wave	Rectangular wave
Amplitude/V	6	6	6	6
Frequency /Hz	0	1k、3k、5k、8k		

*DC* direct current.

Quantitative magnetic flux characterization was performed using the Teslameter under direct current excitation, yielding a magnetic induction intensity of 265.83 mT at a 5 mm air gap between electromagnetic coils. The spatially resolved magnetic field measurements across operational conditions are presented in Table 2.

**Table 2:** Magnetic field strength of electromagnetic coil under different current signals (mT).

Frequency/Hz	DC-SAC	DC-TAC	DC-RAC
1k	402.19	418.48	417.22
3k	452.34	436.58	430.86
5k	424.31	438.92	427.55
8k	455.52	430.37	423.19

*DC* direct current, *SAC* Sine wave alternating current, *TAC* triangular wave alternating current, *RAC* rectangular wave alternating current.

### Test piece preparation

#### Cement mortar

To prepare M20-grade cement mortar specimens, standardized components were adopted, including Portland cement, water, and fine aggregate. Specifically, Type P.O.42.5 ordinary Portland cement was selected as the cementitious material, and its detailed chemical composition is provided in Table 3. For the fine aggregate, river sand was used; prior to application, it was oven-dried at 105 °C and sieved through a 5 mm square mesh to ensure uniform particle size. Meanwhile, laboratory-grade tap water that met the specified requirements served as the mixing medium.

**Table 3:** Cement chemical composition.

Material	Chemical composition (%)							
P.O.42.5	SiO_2_	Fe_2_O_3_	Al_2_O_3_	CaO	MgO	SO_3_	Na_2_O	Loss
	22.32	3.15	5.84	61.23	2.02	2.0	0.15	1.66

The mix proportion of the M20-grade mortar was determined in compliance with the Technical Specification for Mix Proportion Design of Masonry Mortar (JGJ/T 98-2010)^22^, and the specific ratio is listed in Table 4. The mass ratios of cement, sand, and water were first optimized via computational methods, with the final proportions also tabulated in Table 4. All batching processes were carried out systematically under controlled ambient conditions (temperature: 23 ± 2 °C; relative humidity: 50 ± 5%), aiming to minimize the impact of environmental variability on the specimens.

**Table 4:** The M20 mortar mix ratio.

Material	Water	Cement	Fine aggregate
Dosage per m^3^	209 kg	380 kg	1450 kg

#### Sample preparation

**Fig. 3:**
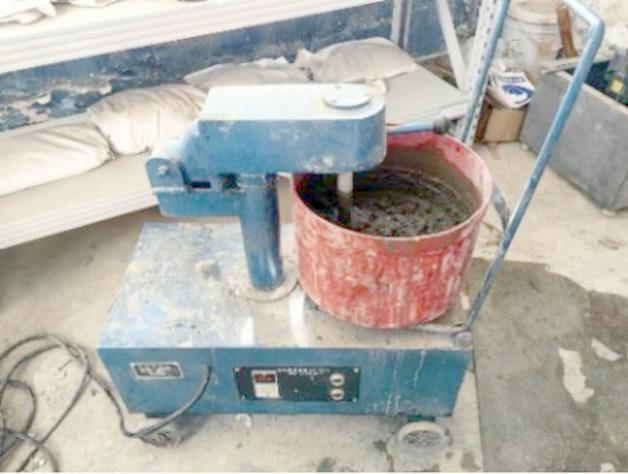
UJZ-15 type mortar mixer.

The cement mortar mixing process was conducted using a UJZ-15 standardized mortar mixer, as illustrated in Fig. 3. A two-stage mixing method was implemented with the objective of optimizing homogeneity, and this was rigorously applied. During the dry blending phase, cementitious constituents and fine aggregates were charged into the mixer bowl at ambient temperature (23 ± 2 °C) and homogenized for 30 s at 140 ± 5 rpm to achieve uniform particle dispersion.

**Fig. 4:**
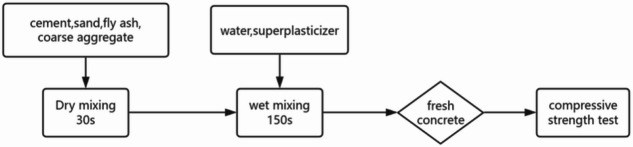
Flow chart of the mixing process.

Subsequent water was then added for wet mixing, with rotational velocity maintained at 285 ± 10 rpm for 150 s. The employment of a two-stage methodology was imperative to ensure complete hydration initiation while minimizing air entrapment. This approach resulted in a total mixing duration of 180 s. The operational workflow was schematically detailed in Fig. 4.

### Test methods and instruments

#### Magnetized water

A thorough quantitative investigation into the properties of magnetized water was conducted in accordance with standardized protocols, employing the following instrumentation and methodologies:

pH measurement: The pH measurements were conducted using a pHS-3 C precision pH meter (with a pH resolution of ± 0.01). Prior to the measurement of the samples, a three-point calibration protocol was implemented. The glass combination electrode was rinsed with triple-distilled water both before and after the measurement in order to prevent cross-contamination.

Electrical Conductivity Analysis: Electrical conductivity was measured using a DDSJ-318 conductivity meter (0-200 mS/cm range, 0.1 µS/cm resolution). The electrode constant was calibrated using a 0.01 mol/L KCl reference solution (1413 µS/cm at 25.0 °C). All measurements were conducted under thermostatically controlled conditions (25.0 ± 0.5 °C), maintained by a recirculating water bath (± 0.1 °C stability). Water samples under each magnetization parameter, 3 repeated tests were conducted to reduce random errors.

Rheological Profiling:

(i) Viscosity measurement: The apparent viscosity was determined using an NDJ-5T rotational viscometer (10 − 2 × 105 mPa s range, LV-3 spindle) under isothermal conditions (25.0 ± 0.1 °C).
(ii) Surface Tension Evaluation: Interfacial tension measurements were conducted using an automated tensiometer (Model ZL-2000 A) with a stated accuracy of ± 0.1 mN/m.

#### Mortar test blocks

**Fig. 5:**
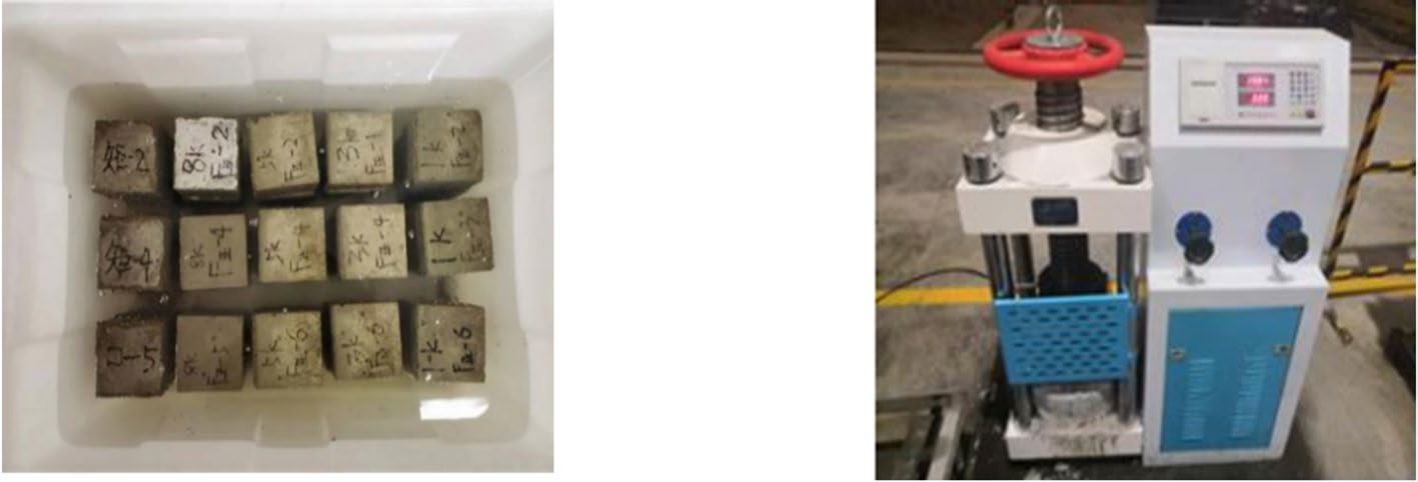
Block curing and compressive strength testing machine.

The curing regime of mortar specimens demonstrated to exert a significant influence on compressive performance development. Figure 5 shows the curing environment and compression testing apparatus.

Standard molds, in accordance with JGJ/T 70-2009 Standard for Test Methods of Basic Properties of Construction Mortar, were employed to contain freshly mixed mortar. Following the initial mold-filling stage, vibratory compaction was performed using an electromagnetic vibration table. The electromagnetic vibration table operated at a frequency of 60 Hz with an amplitude of 0.5 ± 0.05 mm, and the vibratory compaction lasted for 120 ± 5 s. The purpose of this stage of the process was to remove any entrapped air voids. Following the complete solidification of the block, the demolding process should be initiated. Subsequently, the standard curing procedure should be executed at the designated temperature and humidity levels (20 ± 1 °C, RH ≥ 95%).

Compressive strength testing was performed at designated 7d and 28d intervals using a servo-controlled universal testing machine with a displacement rate of 0.5 mm/min. Load-displacement curves were continuously recorded in order to calculate peak stress values and elastic modulus.

### Magnetized water test results

The measurements of pH, conductivity, surface tension and viscosity of tap water treated with different types and frequencies are shown in Tables 5, 6 and 7 below.

**Table 5:** Test results for tap water.

Material	pH	Conductivity C/cm	Surface tension *N*/m	Viscosity mPa.s
Tap water	6.5	129.9	67.6	1.44

**Table 6:** Test results of magnetized water in the noncomposite magnetic field.

Current type	pH		Conductivity C/cm		Surface tension *N*/m		Viscosity mPa s	
orientation	P	S	P	S	P	S	P	S
DC	6.82	7.53	144	158.4	67.2	67.9	1.52	1.48
SAC	7.45	7.85	134.8	161.4	67.2	66.6	1.42	1.53
TAC	7.34	7.74	143.4	162.8	67.3	67.9	1.55	1.55
RAC	7.76	8.07	144.2	165.7	66.9	67	1.59	1.63

*P* parallel arrangement, *S* star arrangement.

**Table 7:** Test results of magnetized water in composite time-varying magnetic field.

Current and frequency	pH		Conductivity C/cm		Surface tension *N*/m		Viscosity mPa s	
Orientation	P	S	P	S	P	S	P	S
DC-SAC1 kHz	7.83	7.88	150.5	165.7	65.8	65.9	1.54	1.55
DC-SAC3 kHz	7.66	7.9	148.3	164.7	65.4	65	1.46	1.63
DC-SAC5 kHz	7.99	8.06	148.1	164.6	64.2	63.9	1.54	1.59
DC-SAC8 kHz	8.11	8.1	149.1	166.4	62	62.3	1.52	1.54
DC-TAC1 kHz	7.71	8.13	149.1	164.1	66.7	65.1	1.44	1.46
DC-TAC3 kHz	7.94	8.25	149.6	165.4	66.5	65.6	1.46	1.57
DC-TAC5 kHz	8.14	8.42	146.9	167.1	66.8	64.5	1.55	1.68
DC-TAC8 kHz	8.08	8.51	147.7	168.7	64	63.3	1.37	1.75
DC-RAC1 kHz	8.01	8.29	149.1	166.1	66	65.1	1.51	1.6
DC-RAC3 kHz	8.15	8.35	148.2	168.4	63.4	64.6	1.46	1.71
DC-RAC5 kHz	8.14	8.47	150.8	171.6	64.3	62.7	1.45	1.79
DC-RAC8 kHz	8.34	8.5	153.6	171.8	63.5	61.8	1.44	1.81

### pH test results

**Fig. 6:**
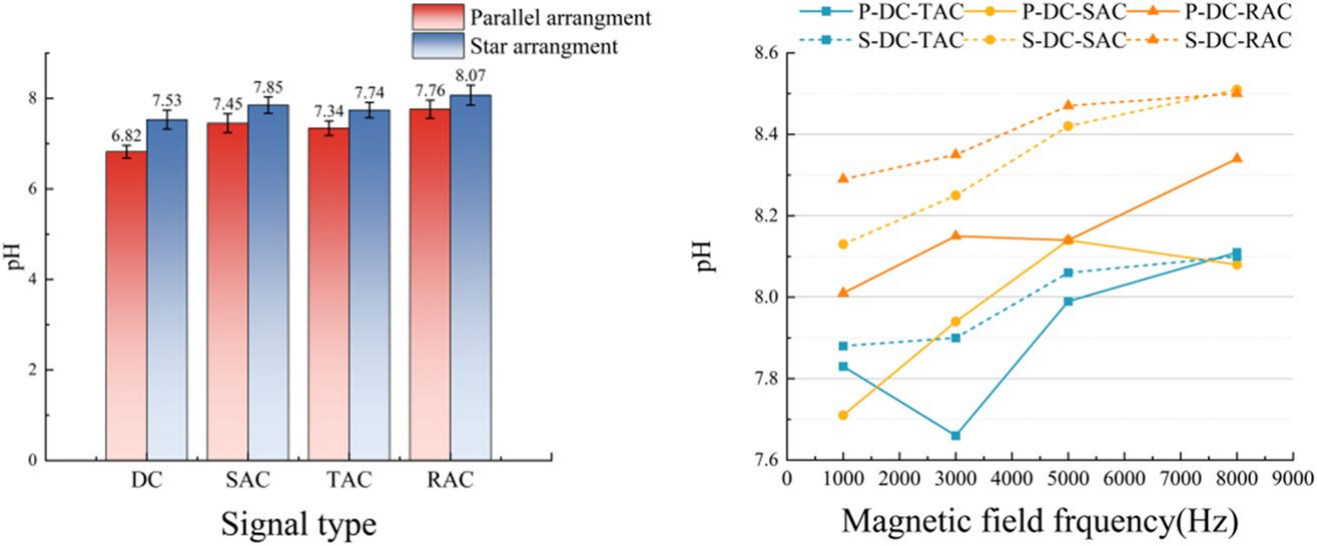
pH test results.

Analysis from Fig. 6 shows that the magnetized water treatment technology can effectively regulate the acid-base characteristics of the water body. With the gradient increase of the magnetic field frequency, the pH value of the water showed a significant growth trend. Under DC-rectangular composite field treatment at 8 kHz, the water pH was elevated from an initial 6.5 to 8.51. Jiang et al.^25^ found that under various magnetization conditions, the pH increase fell within the range of 0.88–1.08. The results showed that the increase in pH was more significant than Jiang’s test. The phenomenon potentially attributed to altered water molecule dissociation processes and enhanced OH^−^ ion mobility^23^.

And significant discrepancies were identified in pH modulation efficiency among different electromagnetic field configurations. The DC-AC composite field exhibited 5.52% and 13.1% greater pH elevation compared to singular AC (pH = 8.07) and pure DC fields (pH = 7.53), respectively. Furthermore, waveform characteristics of composite time-varying electromagnetic fields were found to substantially influence treatment efficacy. The DC-triangular and DC-rectangular wave combinations demonstrated optimal alkalinization performance, with the pH value reaching 8.51. This performance achieved a 5.09% enhancement compared to the DC-sinusoidal wave group, which had a pH value of 8.1. This may originate from the strong gradient electromagnetic perturbation formed by such waveforms during phase switching. The steep waveforms can generate stronger transient vortex electromagnetic fields and promote the directional migration and reorganization of OH^−^ ions^23,24^. This non-stationary electromagnetic environment can effectively induce the spatial rearrangement of charged particles. The magnetized water treatment efficiency can be significantly enhanced by the reasonable design of waveform parameters.

### Conductivity test results

**Fig. 7:**
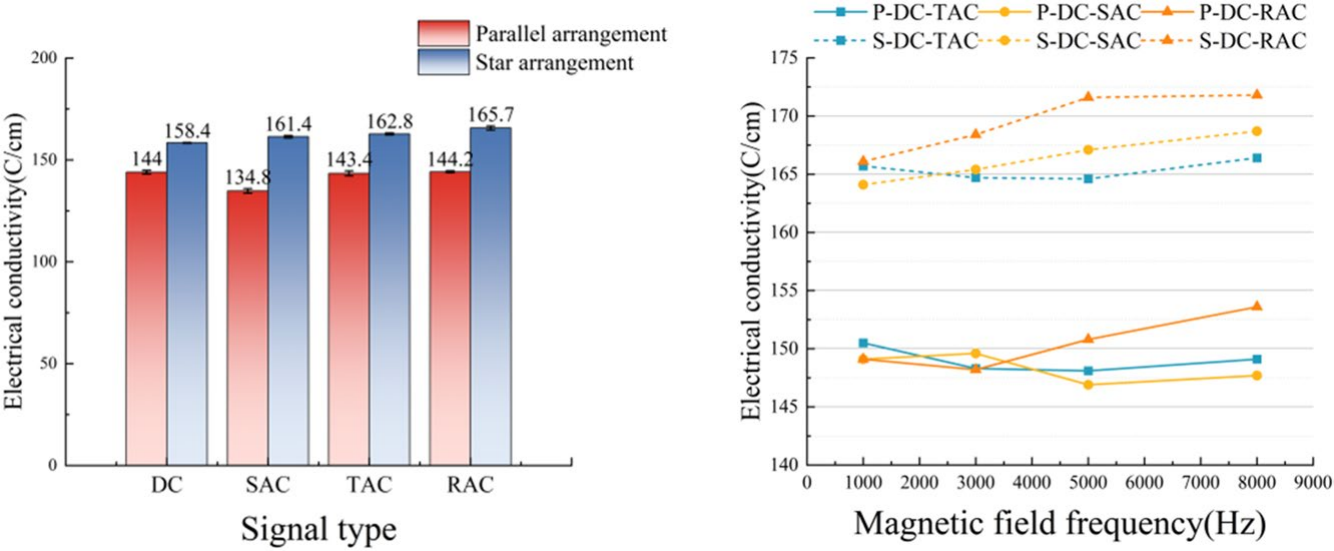
Results of the electrical conductivity test.

As illustrated in Fig. 7, the experimental results clarified the regulatory effect of magnetization treatment on the electrical conductivity of water samples. The data demonstrated a consistent and gradual increase in water conductivity correlating with higher magnetic field frequencies. At a frequency of 8 kHz, the magnetized water sample reached a maximum conductivity of 171.8 µS/cm, representing a 32.3% enhancement compared to untreated samples.

Within time-varying composite field systems, DC-rectangular composite fields demonstrated superior performance compared to DC-triangular and DC-sinusoidal configurations. This superior performance of DC-rectangular composite fields showed consistent correspondence with previously reported pH variation. And magnetic spatial configuration was identified as a critical determinant of treatment efficacy. Experimental measurements indicated that a star-shaped magnetization apparatus achieved 14.2% greater conductivity enhancement than conventional unidirectional configurations. This may originate from the fact that the network of magnetic lines formed by the star arrangement is more favorable for the construction of ion migration channels. During the sudden phase change, the combined waveform electromagnetic field generated a transient strong gradient field. This transient strong gradient field can effectively weaken the hydrogen bonding constraints between water molecules and enhance the kinetic process of ion migration. These effects (weakened hydrogen bonds and enhanced ion migration) thus induce an increase in the conductivity of the water body^26^. The observed interdependence between waveform parameters and conductivity variations emphasizes the importance of electromagnetic field (EMF) optimization in advanced water treatment applications.

### Surface tension test results

**Fig. 8:**
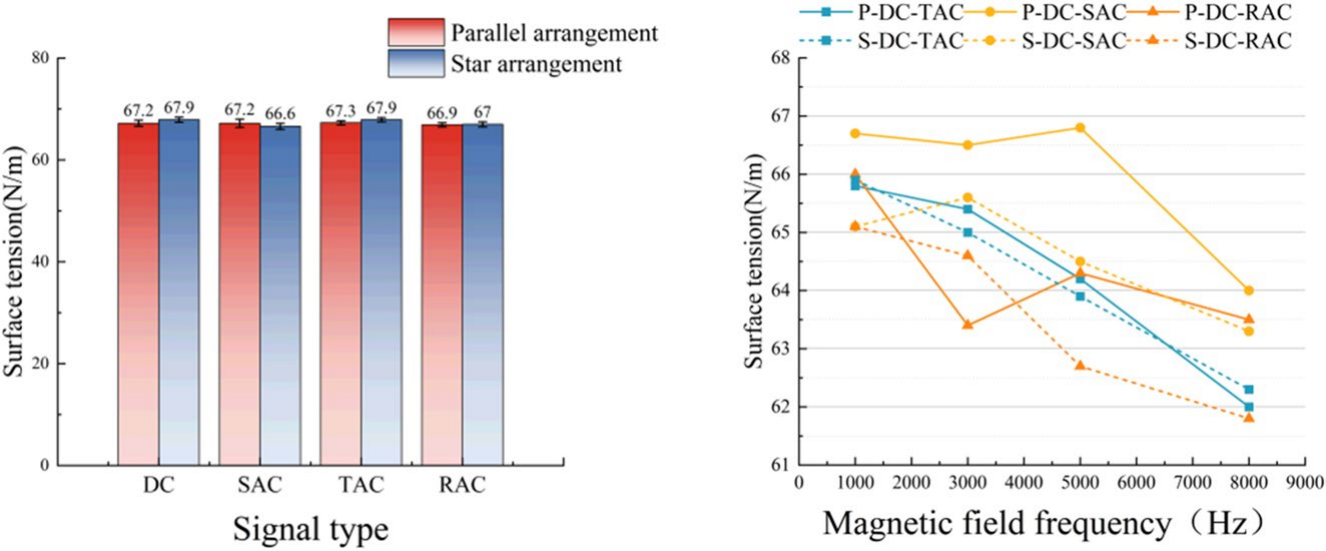
Surface tension test results.

As shown in Fig. 8, the experimental results on surface tension demonstrated a systematic decrease in the surface tension of magnetized water with increasing magnetic field frequency. This conclusion aligned with the findings of Zhao^26^ et al. Zhao^26^ noted the surface tension of magnetized water is more clearly affected by the magnetic field frequency. Within the range from 0 to 800 Hz, the surface tensions of the magnetized and tap water decreased with increasing frequency. The minimum surface tension observed was 61.8 mN/m at 8 kHz, corresponding to an 8.5% reduction compared to untreated samples (67.6 mN/m). Relative to single-frequency AC field (66.6 mN/m) and DC steady-field (67.2 mN/m) treatments, the composite time-varying field group (61.8 mN/m) exhibited reductions of 7.2% and 8.1%, respectively.

From a molecular action mechanism perspective, the alteration in surface tension is predominantly influenced by the equilibrium state of intermolecular force^11^. The experimental data confirmed that magnetic treatment induced a decrease in the surface tension of water, with the surface tension reaching 61.8 N/m(One-way ANOVA, F = 61.52, *P* < 0.001). This surface tension decrease may be attributed to the synergistic effect of high-frequency electromagnetic perturbation and a steady-state magnetic field under the action of a composite time-varying field. This synergistic effect induces the directional arrangement of molecular dipoles, which in turn weakens the intermolecular interaction at the gas-liquid interface^24,27,28^.

### Viscosity test results

**Fig. 9:**
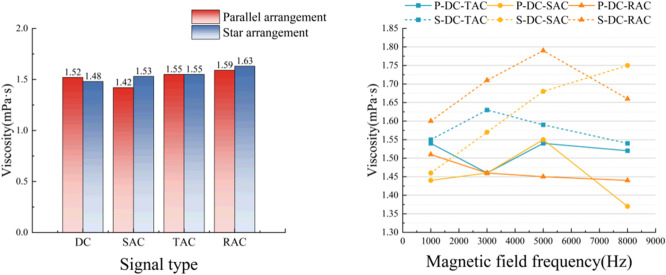
Results of the viscosity test.

As demonstrated in Fig. 9, the viscosity of magnetized water exhibited a non-monotonic response, characterized by an initial increase followed by a subsequent decrease, in response to variations in frequency. The viscosity attained a maximum of 1.79 mPa s under conditions of an 8 kHz magnetic field frequency, representing a 24.3% increase compared to the untreated sample (1.44 mPa s). A comprehensive analysis revealed that the configuration of the electromagnetic field significantly impacted viscosity modulation. The viscosity value of the composite time-varying field treatment group (1.79 mPa s) exhibited an increase of 15.6% and 21.1%, respectively, compared with the single AC field (1.55 mPa s) and the DC steady-state field (1.48 mPa s). This increase was also significantly higher than that observed in the control group (1.44 mPa s) for all the treatment groups.

At the same frequency, the viscosity value of the DC and rectangular wave composite field regime is 1.81 mPa s. This viscosity value increases by 3.4% compared with the DC and triangle wave composite field (1.75 mPa s) and by 17.5% compared with the DC and sinusoidal wave composite field (1.54 mPa s), respectively. This discrepancy may be attributed to the varying degrees of electromagnetic perturbation exhibited by different waveforms. The viscosity of water is essentially determined by the strength of intermolecular forces, and hydrogen bond is the most important interaction force between water molecules. The number, binding strength and dynamic reconstruction characteristics of hydrogen bond directly affect the resistance to the movement of water molecules, and then determine the viscosity of water and its variation law. The external magnetic field acts on the hydrogen bond network of water molecules, causing changes in their structure and dynamic characteristics, and then leading to changes in intermolecular forces (internal friction).

### Test results of cement mortar

The star arrangement of the electromagnetic coil has a better effect on the magnetization of the water than that in the same direction. Therefore, the mortar test is prepared by the star arrangement of the cement mortar. Three specimens of mortar from the same batch and the same age were tested during the compressive strength test.

### Compressive strength

A systematic investigation of mechanical behavior was conducted on mortar specimens subjected to electromagnetic conditioning. The compressive strength of some specimens increased with the increase of frequency, showing a trend of first rising and then decreasing; while the other part decreased first and then increased. Enhanced mechanical performance was consistently recorded in specimens prepared with magnetized water under high-frequency electromagnetic exposure (8 kHz). The maximum 7d compressive strength of the specimens reached 19.39 ± 0.12 MPa when rectangular DC waveforms were applied. This value represents an 11.4% enhancement relative to non-magnetized counterparts (17.41 ± 0.2 MPa). Comparative analysis revealed superior performance in mortar specimens fabricated using water treated by a composite time-varying electromagnetic field. These specimens outperformed those prepared with DC field-magnetized water and AC field-magnetized water by 6.8% and 9.1%, respectively.

**Fig. 10:**
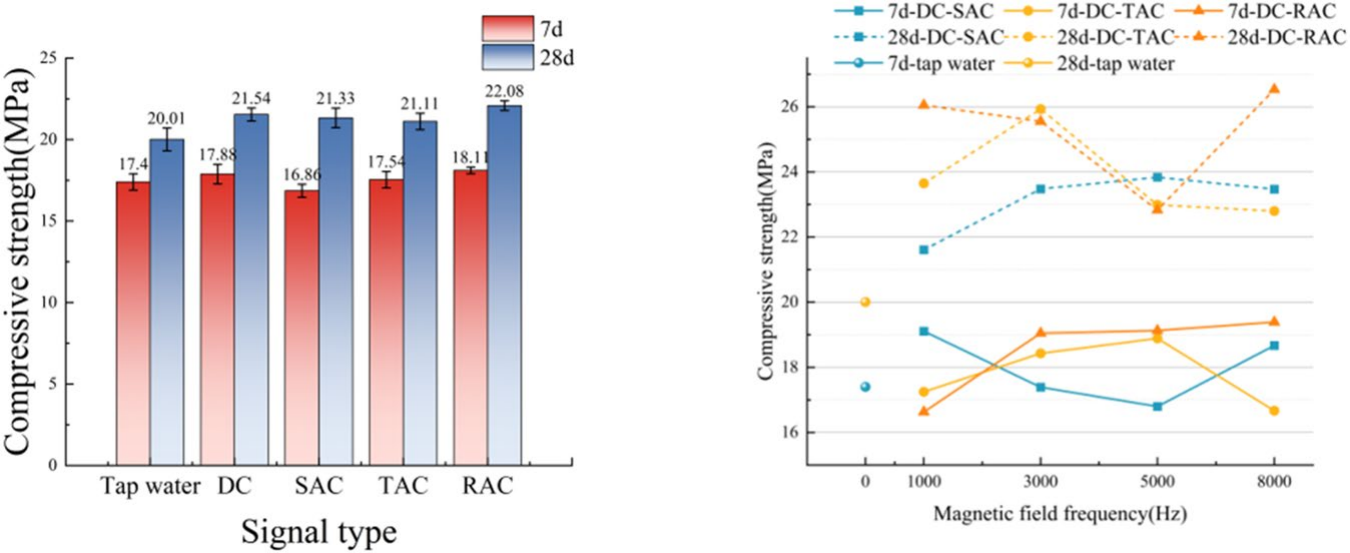
Trend diagram of compressive strength of cement mortar 7d and 28d.

As illustrated in Fig. 10, high-frequency electromagnetic treatment maintained significant advantages for specimens prepared with magnetized water. Under combined DC-triangular wave excitation (1 kHz), the maximum 28d compressive strength of these magnetized water-based specimens reached 26.53 ± 0.31 MPa. This compressive strength corresponds to a 32.5% improvement compared with specimens prepared with conventional water mixtures, which had a compressive strength of 20.03 MPa. Zhao’s study^26^ showed that the maximum 28d compressive strengths at 10, 20, 30, and 40 mT were obtained at 800, 1000, 1200, and 1500 Hz, and the maximum strength increases were 12.3%, 14.8%, 8.4%, and 6.2%, respectively. The technical superiority of composite time-varying electromagnetic protocols was further substantiated, achieving 12.7% and 18.3% greater 28d strength compared to DC and AC electromagnetic treatments, respectively. These findings underscore the technical superiority of composite time-varying electromagnetic protocols in construction material optimization.

### SEM results of cement mortar

**Fig. 11:**
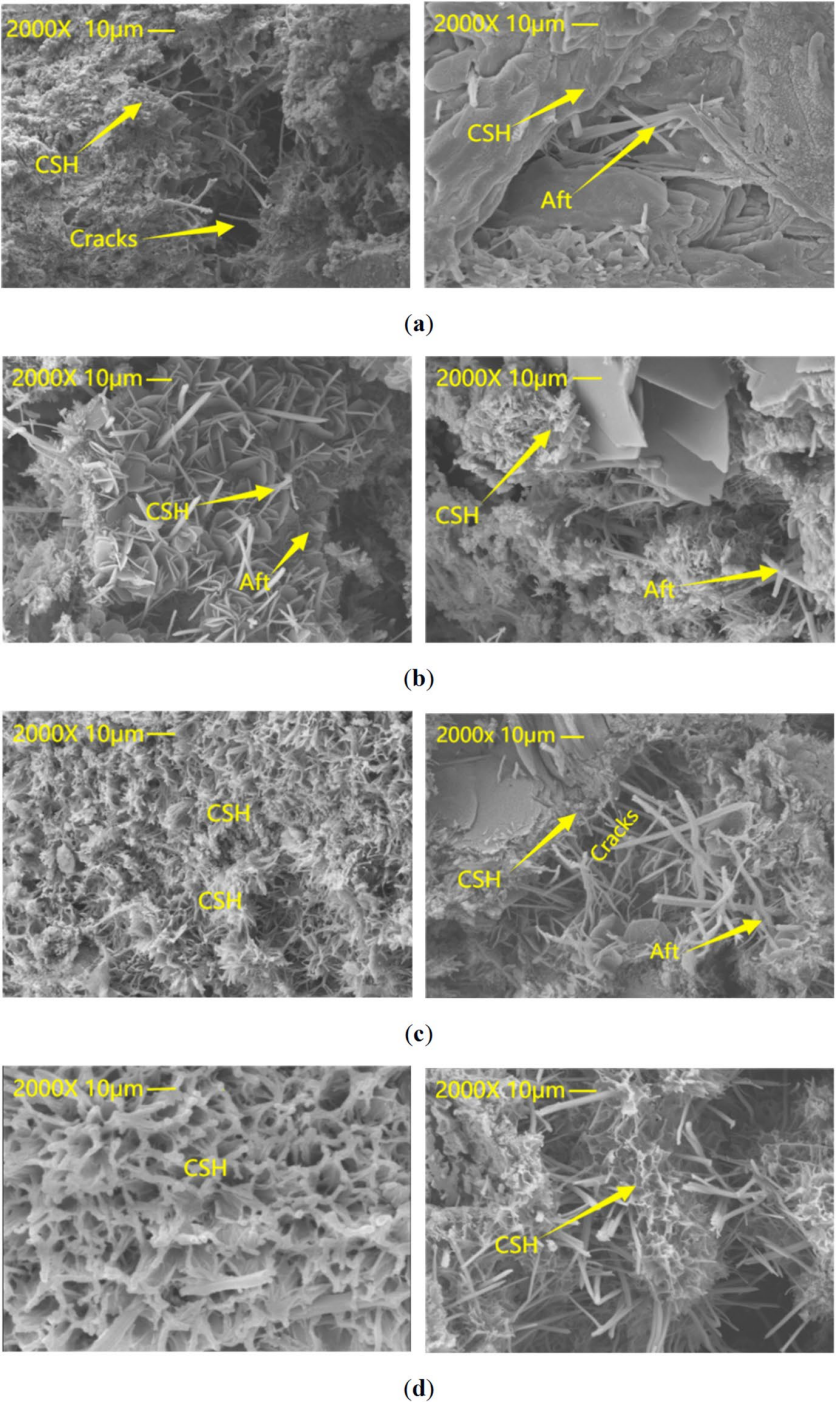
The mixed mortar SEM image of ordinary water and magnetized water at 8 days old. (**a**) Ordinary water mixed with mortar. (**b**) 8 kHz DC-sine wave composite electromagnetic field magnetized water mixed with mortar. (**c**) 8 kHz DC-triangle wave composite electromagnetic field magnetized water mixed with mortar. (**d**) 8 kHz DC-rectangular wave composite electromagnetic field magnetized water mixed with mortar.

A comparative microstructural characterisation of 28d-cured mortar specimens fabricated with conventional and magnetically activated water was systematically performed, as illustrated in Fig. 11. As demonstrated in Fig. 11(a), the surface of the ordinary water-mixed mortar is predominantly constituted by incompletely hydrated granular cement clumps, accompanied by a small number of needle-shaped C-S-H gels. Conversely, the number of granular agglomerations on the surface of magnetized water-mixed mortar was reduced, and flocculating C-S-H gel became more obvious. The microstructure of mortar mixed with water using a composite time-varying electromagnetic field is demonstrated in (b), (c) and (d) of Fig. 11.The surface of the material is composed of fibrous mesh, with a significant number of columnar products present between the gaps. These products possess intricate structural compositions, characterized by interwoven layers and substantial columnar formations. This observation indicates that the hydration reaction of cement particles in this case is more vigorous than in Fig. 11 (a). The rate of C-S-H gel generation is faster, the distribution is more uniform and even, and the C-S-H gels growing in a staggered pattern within the cracks effectively fill the gaps.

As illustrated in Fig. 11a, the presence of layered structures and a reduced amount of fiber C-S-H gels is evident. The outcomes of the compressive strength test indicate a low level of strength, thereby substantiating the correlation between microstructural characteristics and macroscopic performance metrics. As illustrated in Fig. 11b–d, the substantial density of particles and the compactness of the structure are of considerable significance. The magnetized water has been shown to promote the formation of hydrated calcium silicate (C-S-H) gels. The formation of a dense structure during the hardening process is attributed to the gel, which has been demonstrated to effectively improve microscopic defects, including cracks and holes. Consequently, this results in an enhancement of the material’s overall performance. SEM analysis (Fig. 11) demonstrated that the C-S-H gel in the magnetized water group exhibited an interwoven fibrous structure (ext-diameter ratio > 10), and the porosity was reduced by 37.2% in comparison with the control group. In the microstructure of magnetized water mortar prepared by composite electromagnetic fields, tentacle-shaped AFt crystals grow optimally along the direction of the magnetic field, forming a three-dimensional reinforcement framework. This results in an improvement of the compressive strength of the mortar.

## Conclusions

1. The experimental results demonstrate that the pH and conductivity of magnetized water increase gradually with higher magnetic field frequency, while surface tension decreases with increasing frequency. Viscosity of magnetized water increased initially then decreased slightly.
2. When treated with a 5 kHz DC sinusoidal combined electromagnetic field, the 7d compressive strength of cement mortar increased by up to 11.4%. The DC-triangular combined electromagnetic field (1 kHz) exhibited a substantial 32.5% enhancement in 28d compressive strength.
3. SEM analysis confirmed that water treated with composite electromagnetic fields showed a superior capacity to reduce microscopic defects in cement mortar compared to exposure to singular AC or DC magnetic fields.
4. This paper only studies the compressive strength of M20 mortar. Future studies need to further explore the effects of composite time-varying electromagnetic fields on the performance of other types of cement-based materials, and evaluate their engineering applicability.

## Data Availability

All data generated or analyzed during this study are included in this published article.

## References

[1] Ding, Y. H. et al. *Bull. Chin. Ceramic Soc.*, **40**, 1178–1185. 10.16552/j.cnki.issn1001-1625.2021.04.014 (2021).

[2] Ahmed, H. I. Behavior of magnetic concrete incorporated with Egyptian nano alumina. *Constr. Build. Mater.* **150**, 404–408. 10.1016/j.conbuildmat.2017.06.022 (2017).

[3] Mitrofanov, V. V., Romanovsky, Y. M. & Netrebko, A. V. On the structure and dynamics of hydrogen bonds in liquid water (2004).

[4] Henchman, R. H. Water’s dual nature and its continuously changing hydrogen bonds. *J. Phys.: Condens. Matter*. **28**, 384001 (2016). 27447299 10.1088/0953-8984/28/38/384001

[5] Pang, X. F. & Deng, B. The changes of macroscopic features and microscopic structures of water under influence of magnetic field. *Phys. B: Condens. Matter*. **403**, 3571–3577. 10.1016/j.physb.2008.05.032 (2008).

[6] Mu, L. H. *Theoretical Study on the Effects of Water Molecules on the Properties of Several carbon-based Nanomaterials Via Hydrated cation-π Interactions* (University of Chinese Academy of Sciences (Shanghai Institute of Applied Physics,, 2022). Chinese Academy of Sciences).

[7] Ding, Z. R., Zhao, Y., Chen, J., Chen, F. L., Duan, S. & J. Z. & X. Magnetization mechanism of magnetized water. *Acta Phys. Sin*. **60** (6), 064701. 10.7498/aps.60.064701 (2011).

[8] Iwasaka, M. & Ueno, S. Structure of water molecules under 14 T magnetic field. *J. Appl. Phys.* **83**, 6459–6461. 10.1063/1.367737 (1998).

[9] Ebrahimi Jouzdani, B. & Reisi, M. Effect of magnetized water characteristics on fresh and hardened properties of self-compacting concrete. *Constr. Build. Mater.* **242**, 118196. 10.1016/j.conbuildmat.2020.118196 (2020).

[10] Demir, I., Alakara, E. H., Sevim, O. & Kartal, S. Effect of magnetized water on alkali-activated slag mortars incorporating Raw and calcined marble powder. *Constr. Build. Mater.* **424**, 135943. 10.1016/j.conbuildmat.2024.135943 (2024).

[11] Hamed, Y. R., Yousry Elshikh, M. M., Elshami, A. A., Matthana, M. H. S. & Youssf, O. Mechanical properties of fly Ash and silica fume based geopolymer concrete made with magnetized water activator. *Constr. Build. Mater.* **411**, 134376. 10.1016/j.conbuildmat.2023.134376 (2024).

[12] Hu, H. X. & Deng, C. Effect of magnetized water on the stability and consolidation compressive strength of cement Grout. *Materials* **14** (2021). 10.3390/ma14020275 PMC7828106 33430416

[13] Wang, Y. K. & Wei, H. N. Effect and analysis on performance of compression and tension of magnetized water concrete with different magnetic courses. *Sci. Technol. Eng.* **16**, 144–148 (2016).

[14] Ghorbani, S., Ghorbani, S., Tao, Z., de Brito, J. & Tavakkolizadeh, M. Effect of magnetized water on foam stability and compressive strength of foam concrete. *Constr. Build. Mater.* **197**, 280–290. 10.1016/j.conbuildmat.2018.11.160 (2019).

[15] Yesin, K. B., Vollmers, K. & Nelson, B. J. Modeling and control of untethered biomicrorobots in a fluidic environment using electromagnetic fields. *Int. J. Robot. Res.* **25**, 527–536 (2006).

[16] Jeong, S. et al. Novel electromagnetic actuation (EMA) method for 3-dimensional locomotion of intravascular microrobot. *Sens. Actuators A: Phys.* **157**, 118–125 (2010).

[17] Sikorski, J., Dawson, I., Denasi, A., Hekman, E. E. G. & Misra, S. Introducing BigMag — A novel system for 3D magnetic actuation of flexible surgical manipulators. In *IEEE International Conference on Robotics and Automation (ICRA)*. 3594-3599.3 (2017).

[18] Liang, X. & Li, Q. Application effect of variable frequency magnetized water irrigation technology. *Agricultural Eng.* **3**, 43–44 (2013).

[19] Wang, Y. K. & Wei, H. N. Study on influence of frequency-converted magnetized water on crack resistance of concrete. *Building Struct.* **48**, 107–111. 10.19701/j.jzjg.2018.22.018 (2018).

[20] Jiang, S. *The Theory and Key Technologies of the Pulsed High Magnetie Field with a High Repetition Rate* (Huazhong University of Science and Technology, 2022).

[21] Li, K. K. et al. Effect of pulsed magnetic field frequency on scaling process of CaCO_3_ solution. *J. Inner Mongolia Univ. Sci. Technol.* **41**, 194–198. 10.16559/j.cnki.2095-2295.2022.02.016 (2022).

[22] Code for mix design of masonry mortar. Vol. JGJ/T 98-2010 38P.;B35. Industry Standard-Construction industry. (2010).

[23] Ghauri, S. A. & Ansari, M. S. Increase of water viscosity under the influence of magnetic field. *J. Appl. Phys.* **100**, 066101. 10.1063/1.2347702 (2006).

[24] Rong, X. et al. Characteristics,Mechanism and applications of magnetized water:a review. *Mater. Rep.* **36**, 65–71 (2022).

[25] Jiang, H. et al. Effect of magnetization conditions on the slump and compressive strength of magnetized water concrete. *Case Stud. Constr. Mater.* **21**, e03920. 10.1016/j.cscm.2024.e03920 (2024).

[26] Zhao, G., Zhang, Z., Ma, N., Wang, Y. & Cheng, S. Preparation and characterization of cement mortar mixed with alternating field-magnetized water. *Constr. Build. Mater.* **416**, 135204. 10.1016/j.conbuildmat.2024.135204 (2024).

[27] Toledo, E. J. L., Ramalho, T. C. & Magriotis, Z. M. Influence of magnetic field on physical–chemical properties of the liquid water: insights from experimental and theoretical models. *J. Mol. Struct.* **888**, 409–415. 10.1016/j.molstruc.2008.01.010 (2008).

[28] Su, N. & Wu, C. F. Effect of magnetic field treated water on mortar and concrete containing fly Ash. *Cem. Concr. Compos.* **25**, 681–688. 10.1016/S0958-9465(02)00098-7 (2003).

